# Aquariums as Reservoirs for Multidrug-resistant *Salmonella* Paratyphi B

**DOI:** 10.3201/eid1203.051085

**Published:** 2006-03

**Authors:** Renee S. Levings, Diane Lightfoot, Ruth M. Hall, Steven P. Djordjevic

**Affiliations:** * Elizabeth Macarthur Agricultural Institute, Camden, New South Wales, Australia;; † University of Wollongong, Wollongong, New South Wales, Australia;; ‡ University of Melbourne, Melbourne, Victoria, Australia;; § University of Sydney, Sydney, New South Wales, Australia

**Keywords:** Salmonella, ornamental fish tanks, multiple antimicrobial drug resistance, dispatch

## Abstract

Multidrug-resistant *Salmonella enterica* serovar Paratyphi B dT+ isolates from patients with gastroenteritis were identical with isolates from their home aquariums. Matched isolates had identical phage types, *Xba*I and IS*200* profiles, and *Salmonella* genomic island 1 (SGI1). Ornamental fish tanks are reservoirs for SGI1-containing *S*. Paratyphi B dT+.

Strains of *Salmonella enterica* serovar Paratyphi B that use d-tartrate as a carbon source (*S*. Paratyphi B dT+, formerly *S*. *enterica* serovar Java) primarily cause gastroenteritis (*1*). Since the late 1990s, multidrug-resistant *S*. Paratyphi B dT+ has been increasingly isolated from infected persons in different parts of the world. One type, which is resistant to streptomycin, spectinomycin, trimethoprim, and sulfonamides, carries a chromosomally located class 2 integron with the *dfrA1*-*sat1*-*aadA1* (Tn*7*) array of gene cassettes (*2*). This clone is predominantly associated with poultry and poultry products in Germany and the Netherlands (*2**,**3*). Human cases of gastroenteritis caused by *S*. Paratyphi B dT+ with the resistance phenotype ApCmSmSpSuTc (Ap, ampicillin; Cm, chloramphenicol; Sm, streptomycin; Sp, spectinomycin; Su, sulfonamides; Tc, tetracycline) have also been found in Canada (*4*), the United Kingdom (*5*), France (*6*), and Australia (*7*), and their incidence is increasing. In most of the studied isolates, the resistance genes *blaP1*, *floR*, *aadA2*, *sul1*, and *tetG* are located in a complex class 1 integron recently designated In104 (*7*) (Figure 1). This integron is located within the *Salmonella* genomic island 1 (SGI1) that was first identified in *S. enterica* serovar Typhimurium DT104 strains with the same phenotype (*8*). However, the source of the SGI1-containing *S*. Paratyphi B dT+ has not been identified. Whether isolates obtained in different countries are clonally related is also not known.

**Figure 1:**
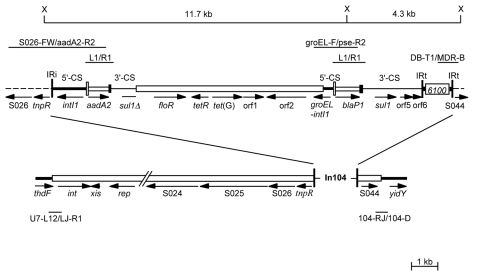
Structure of *Salmonella* genomic island 1 (SGI1). The SGI1 region of serovar Typhimurium DT104 (GenBank accession no. AF261825) is drawn to scale with In104 above. Different discrete segments are represented by open boxes and lines of different thicknesses. Vertical bars indicate the IR bounding In104. The chromosomal genes *thdF* and *yidY* flank SGI1. Fragments amplified by polymerase chain reaction are shown as thin lines with the primer pairs indicated. Sequences of primers have been previously described (*7*). Arrows indicate the position and orientation of genes and open reading frames. The positions of *Xba*I sites (×) are indicated with the fragment sizes shown.

Although a few epidemiologic studies suggest that antimicrobial drug–susceptible *S*. Paratyphi B dT+ may be linked to aquacultural practices (*9**,**10*), no molecular data confirm this. However, the first reported SGI1-containing *S*. Paratyphi B dT+ isolate with drug-resistance phenotype ApCmSmSpSuTc was isolated in 1997 from a tropical fish in Singapore (*11*), raising the possibility that tropical fish and aquariums are a reservoir. The aim of this study was to determine if domestic aquariums are reservoirs for SGI1-containing, multidrug-resistant *S*. Paratyphi B dT+ that infect humans.

## The Study

*S*. Paratyphi B dT+ with the resistance phenotype ApCmSmSpSuTc had been isolated sporadically in various states of Australia since 1997, and initial surveys showed a potential association with ownership of home aquariums (D. Lightfoot, unpub. data). In 2000, multidrug-resistant *S*. Paratyphi B dT+ with an identical phage type (reaction does not conform [RDNC]), designated here as Aus2, and the same drug-resistance profile (ApCmSmSpSuTc) was isolated from humans with gastroenteritis and from fish tanks in the homes of 2 infected patients (Table). In 2003 and 2004, 13 cases of ApCmSmSpSuTc *S*. Paratyphi B dT+ were investigated by state and commonwealth health departments, and all were associated with home aquariums containing tropical fish (J. Musto et al., unpub. data). Of these, 11 cases were phage type RDNC Aus3, 1 was phage type 1 var 15, and 1 was phage type 3b var. Water and gravel were collected from the domestic aquariums of 5 patients with RDNC Aus3-type infections, and identical isolates were recovered from each fish tank. Four matched sets of isolates, 2 from 2000 and 2 from 2003, were further examined (Table). One isolate (SRC50) characterized previously (*7*) was used as a control (Table).

**Table:** Genetic characteristics of *Salmonella enterica* serovar Paratyphi B dT+ isolates used in this study

Isolate no.*	Source†	Phage type‡	State§	Date of isolation	Age, y/Sex	SGI1¶
Set 1						
SRC229	H	Aus2	ACT	2000	<1/F	+
SRC230	H	Aus2	ACT	2000	1/M	+
SRC231	FT	Aus2	ACT	2000	–	+
Set 2						
SRC232#	H	Aus2	Vic	2000	11/F	+
SRC233#	H	Aus2	Vic	2000	11/F	+
SRC233A	FT	Aus2	Vic	2000	–	ND
Set 3						
SRC145	H	Aus3	Vic	2003	74/F	+
SRC142	FT	Aus3	Vic	2003	–	+
SRC143	FT	Aus3	Vic	2003	–	+
Set 4						
SRC149	H	Aus3	Vic	2003	12/M	+
SRC147	FT	Aus3	Vic	2003	–	+
SRC148	FT	Aus3	Vic	2003	–	+
Control						
SRC50	H	RDNC	Vic	2001	14/M	+**

*All isolates were resistant to ampicillin, chloramphenicol, streptomycin, spectinomycin, sulfonamides, and tetracycline. †H, human isolates; FT, fish tank isolates. ‡Determined by using standard procedures and designations (http://www.geocities.com/avinash_abhyankar/typing.htm). RDNC, reaction does not conform. RDNC Aus2 and RDNC Aus3 are 1 var and 3b var phage-typing variants, respectively, and are identifiable and reproducible phage-typing patterns awaiting formal assignment by the World Health Organization–designated International Reference Laboratory, Colindale, UK. §ACT, Australian Capital Territory; Vic, Victoria. ¶SGI1, *Salmonella* genomic island 1; ND, strain not available for molecular analysis. #Isolates are from the same person. **Data from Levings et al. (*7*).

To determine if the resistance phenotype of these strains was due to SGI1 (*4**,**5**,**7**,**8**,**11*), polymerase chain reaction (PCR) with primer pairs shown in Figure 1 was used as previously described (*7*). The left and right junctions of SGI1 with the chromosome and of In104 with SGI1 were present in all cases. Regions containing the gene cassettes were amplified by using standard primers (L1 and R1) in the 5´- and 3´-conserved segments of class 1 integrons. Fragments of 1.0 and 1.2 kb were amplified from all isolates, and digestion of these amplicons with *Rsa*I generated a profile (data not shown) that was indistinguishable from the pattern for the 2 amplicons containing the *aadA2* and *blaP1* cassettes found in In104 and *S*. Paratyphi B dT+ isolates SRC49 and SRC50 from 2001 (*7*). The *aadA2* gene cassette was linked to SO26 in the SGI1 backbone, which indicates that it is on the left, as in In104, and the expected 1.8-kb PCR fragment was generated by using primers in *groEL* and *blaP1* (Figure 1), which places the *blaP1* cassette on the right. Southern hybridization of *Xba* I–digested whole-cell DNA with a probe for the *floR* gene as described previously (*7*) identified a band of ≈12 kb, which is consistent with an SGI1 structure identical to that reported previously (*7**,**8**,**11*) and the *groEL*-*blaP1* amplicon linked this 12-kb *Xba*I fragment with the adjacent 4.3-kb *Xba*I fragment (Figure 1).

**Figure 2:**
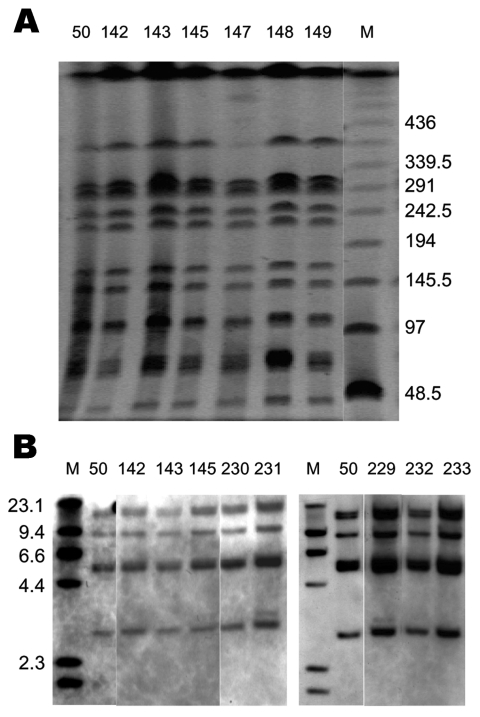
Pulsed-field gel electrophoresis (PFGE) and IS*200* profiles of *Salmonella enterica* serovar Paratyphi B dT+ isolates positive for *Salmonella* genomic island 1. A) PFGE profiles. *Xba* I–digested whole-cell DNA was separated by PFGE as previously described (*12*). Molecular mass markers (lane M) are low-range PFGE markers (New England BioLabs, Beverly, MA, USA) composed of concatamers of bacteriophage lambda DNA. The band absent in lane 147 was present in other runs. B) IS*200* profiles. *Pst*I digests of whole-cell DNA were separated and hybridized with an IS*200* digoxigenin (DIG)–labeled probe. Molecular mass markers (lane M) are DIG-labeled bacteriophage lambda DNA digested with *Hind*III (Roche Diagnostics, Castle Hill, New South Wales, Australia). Primers and polymerase chain reaction conditions used to generate the IS*200* probe have been previously described (*6*).

To obtain further evidence for the identity of the matched human and fish tank isolates, macrorestriction analyses of *Xba*I–digested whole-cell DNA by pulsed-field gel electrophoresis (PFGE) were performed as previously described (*12*). Several studies (*3**–**6**,**13*) suggest that *S*. Paratyphi B dT+ isolates possess considerable genetic heterogeneity. However, the SGI1-containing isolates appear to be homogeneous. The band patterns for all SGI1-containing *S*. Paratyphi B dT+ were identical from humans and fish tanks with phage type RDNC Aus3 (Figure 2A) and Aus2 (data not shown). IS*200* profiles were also analyzed by hybridization of an IS*200* probe with *Pst* I–digested whole-cell DNA as described elsewhere (*6*). Again, all strains showed identical profiles (Figure 2B and data not shown) that differed by 1 band from profile IP1 recently described (*6*). Thus, matched isolates from humans and their fish tanks were indistinguishable from each other.

An unusual observation in this study was that isolates with different phage types showed identical PFGE and IS*200* profiles, indicating that they represented a clonal cluster. The control strain SRC50 (RDNC) also displayed the same patterns, demonstrating that it also is a member of the same clone. Thus, variation in phage type (Table) appears to have occurred within a single clone. Variation in phage type has also been reported in other studies of multidrug-resistant *S*. Paratyphi B dT+ strains (*4**–**6*), although a number of related but slightly different *Xba*I PFGE patterns were observed in those studies. This finding suggests that all multidrug-resistant *S*. Paratyphi B dT+ found globally have a single origin, but that variations, possibly because of acquisition of other temperate phages or plasmids, have arisen over time. However, direct comparisons of strains from different countries will be needed to confirm this hypothesis.

## Conclusions

This is the first definitive report showing that ornamental fish tanks are a reservoir for multidrug-resistant *S*. Paratyphi B dT+ (ApCmSmSpSuTc phenotype) containing SGI1 that causes severe disease in humans, particularly young children. In addition to containing SGI1, the matched isolates from humans and their fish tanks had the same phage type and the same *Xba*I macrorestriction digest pattern and IS*200* profile. These findings identify home aquariums containing tropical fish as the most important, although not necessarily the only, source of multidrug-resistant *S*. Paratyphi B dT+. The fact that 12%–14% of Australian households have ornamental fish (*14*) and as many as 12 million American and 1 million Canadian families own domestic aquariums (*9*), together with the young age of most affected patients, indicate that multidrug-resistant *S*. Paratyphi B dT+ in home aquariums is a risk factor for *Salmonella* infection and thus becomes a public health issue.
